# Association between decreased serum TBIL concentration and immediate memory impairment in schizophrenia patients

**DOI:** 10.1038/s41598-018-38227-6

**Published:** 2019-02-07

**Authors:** Xiao Li Yin, Qiu Fang Jia, Guang Ya Zhang, Jian Ping Zhang, Tomoaki Shirao, Cai Xia Jiang, Xu Yuan Yin, Yan Song Liu, Peng Chen, Xiao Chu Gu, Zheng Kang Qian, Guang Zhong Yin, Hai Sen Xia, Li Hui

**Affiliations:** 1 Wenzhou Kangning Hospital, Wenzhou Medical University, Zhejiang, China; 2 Institute of Mental Health, The Affiliated Guangji Hospital of Soochow University, Soochow University, Jiangsu, China; 3 Suzhou Center for Disease Prevention and Control, Jiangsu, China; 4 Department of Psychiatry, Donald and Barbara Zucker School of Medicine at Hofstra/Northwell, Hempstead, East Garden City, NY USA; 5 Division of Psychiatry Research, The Zucker Hillside Hospital, Northwell Health, Glen Oaks, NY USA; 6 Department of Neurobiology and Behavior, Graduate School of Medicine, Gunma University, Maebashi, Japan; 7 Mengcheng Brain Health Hospital, Anhui, China

## Abstract

Cognitive impairment is a core feature of schizophrenia (SCH). In addition to the toxic effect of Bilirubin (BIL), it has antioxidant properties that were associated with the psychopathology and cognitive impairment of psychiatric disorders. The aim of this study was to examine the correlation of serum total BIL (TBIL) concentration with cognitive impairment in SCH patients. We recruited 34 SCH patients and 119 healthy controls (HCs) in this case-control design. Cognition was assessed using the Repeatable Battery for the Assessment of Neuropsychological Status (RBANS). Serum TBIL concentration was measured using the immunoturbidimetric method. Serum TBIL concentration was significantly decreased in SCH patients compared to HCs after adjusting for age, gender, and education. Serum TBIL concentration in SCH patients was also positively correlated with the RBANS immediate memory score. Further stepwise multiple regression analysis confirmed the positive association between serum TBIL concentration and immediate memory score in SCH patients. Our findings supported that the decline in serum TBIL concentration was associated with the immediate memory impairment and psychopathology of SCH.

## Introduction

Schizophrenia (SCH) is a severe psychiatric disorder that affects around 0.7% of the population worldwide^1^. Although SCH primarily involves a series of psychiatric symptoms, cognitive impairment is regarded as a core feature of SCH that precedes the onset of psychotic symptoms_,_ and persists throughout the illness course^2–6^. SCH-associated cognitive impairment emerges in almost all cognitive domains_,_ and may influence treatment outcomes, rehabilitation, quality of life, and even employment^7–14^. Collectively, these findings support the notion that cognitive impairment should be considered a potential treatment target for SCH and an important focus of future studies. However, the underlying pathophysiology of cognitive impairment in SCH patients remains unclear and requires further investigation.

Bilirubin (BIL) is a powerful antioxidant that is believed to stem from heme^15,16^. It has been reported that BIL demonstrates a strong protective function in the body when the mechanism of defense against oxidative stress is challenged^17^. Interestingly, both increased and decreased total BIL (TBIL) concentration (or a diversity of BIL-related effects) associated with SCH patients has been reported in previous studies. For example, several studies have shown that plasma TBIL concentration in SCH patients is significantly decreased compared to healthy controls (HCs)^18–22^. Similarly, plasma TBIL concentration in first-episode SCH patients is found to be significantly lower than that in HCs^22^. Further study has indicated that both male and female SCH patients have an approximately two-fold lower BIL concentration compared to HCs^23^. Moreover, the urinary concentration of biopyrrins generated from BIL in SCH patients is significantly higher than that in HCs^24,25^. These studies support the hypothesis that the antioxidant system defects exist in SCH patients^26,27^. However, two recent studies have indicated that serum BIL concentration in SCH patients is significantly elevated compared to HCs^28,29^. Also, previous studies have shown that there is a significant association between moderate hyperbilirubinemia and SCH^24,30–34^. Therefore, further studies should be conducted to resolve the inconsistency among these findings.

Previous studies have shown that abnormal BIL concentration may influence cognitive function. For example, serum BIL concentration is reported to be positively correlated with the Mini-Mental State Examination scores and Montreal Cognitive Assessment (including attention, delayed recall and abstract) in patients with mild cognitive impairment (MCI)^35^. Another study finds a positive correlation between serum BIL concentration and cognitive function originated from a great number of cognitive domains in patients with subcortical ischemic vascular disease (SIVD)^36^. Abnormal concentration of BIL in the brain can cause microglia and astrocyte activation, impaired myelination, and neuronal cell death^37^. Moreover, serum TBIL concentration in Korean adults is closely associated with the leukoaraiosis, which is relevant to cognitive impairment^38–41^. These findings indicate that serum TBIL concentration may influence cognitive function in SCH patients. However, to our knowledge, no study has investigated the relationship between serum TBIL concentration and cognitive impairment in SCH patients. Therefore, the objective of this study was to examine whether serum TBIL concentration is significantly associated with cognitive impairment in SCH patients in a Chinese population.

## Results

### Sociodemographic and clinical characteristics

Table 1 showed the sociodemographic and clinical characteristics of all subjects. SCH patients and HCs did not differ in age, gender, and education. There were significant differences in the Repeatable Battery for the Assessment of Neuropsychological Status (RBANS) total score and subscores of immediate memory, attention, language, and delayed memory between the two groups. These between-group differences remained significant after adjusting for age, gender and education. These significant cognitive differences also passed the Bonferroni correction. Further Pearson’s correlation analysis found that serum TBIL concentration was not related to the Positive and Negative Syndrome Scale (PANSS) total score, PANSS negative symptom, PANSS positive symptom, and PANSS general psychopathology in SCH patients.

**Table 1:** The sociodemographic and clinical characteristics in SCH patients and HCs.

Variables	SCH Patients (n = 34)	HCs (n = 119)	*P* Value
Sex (male/female)	21/13	71/48	0.83
Age (years)	47.74 ± 10.02	45.71 ± 9.93	0.30
Education (years)	10.88 ± 3.12	11.63 ± 3.94	0.31
Age of Onset (years)	25.03 ± 7.14		
PANSS-Positive Symptom	12.06 ± 4.53		
PANSS-Negative Symptom	18.03 ± 8.03		
PANSS-General Psychopathology	26.68 ± 6.75		
PANSS-Total Score	56.76 ± 15.85		
RBANS-Immediate Memory	68.88 ± 18.72	93.03 ± 15.66	3.22 × 10^**−12**^
RBANS-Attention	87.03 ± 15.45	112.86 ± 15.99	3.67 × 10^**−14**^
RBANS-Language	85.71 ± 12.91	98.21 ± 12.60	1.12 × 10^**−6**^
RBANS-Visuospatial/Constructiona	89.71 ± 19.54	86.01 ± 13.27	0.90
RBANS-Delayed Memory	77.79 ± 16.49	94.35 ± 7.74	6.84 × 10^**−14**^
RBANS-Total Score	76.97 ± 14.55	91.41 ± 14.17	2.73 × 10^**−13**^

Note: Mean ± SD, SCH = schizophrenia; Healthy controls = HCs; PANSS = Positive and negative syndrome scale.

### Serum TBIL concentration between SCH patients and HCs

**Figure 1:**
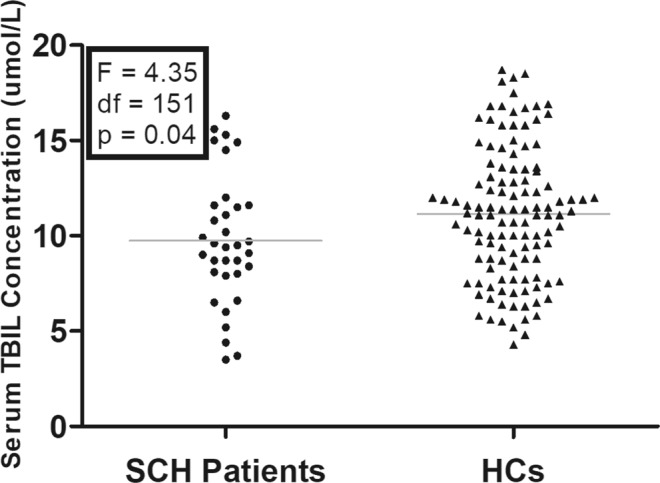
Comparison of serum TBIL concentration between SCH patients and HCs. Serum TBIL concentration was significantly lower in SCH patients than that in HCs (9.74 ± 3.38 vs. 11.14 ± 3.49 umol/L, F = 4.35, p = 0.04). Abbreviations: SCH, schizophrenia; HCs, healthy controls.

Figure 1 showed that serum TBIL concentration in SCH patients was significantly decreased compared to HCs. This between-group difference remained significant after adjusting for age, gender and education.

### Associations of serum TBIL concentration with the RBANS scores

**Figure 2:**
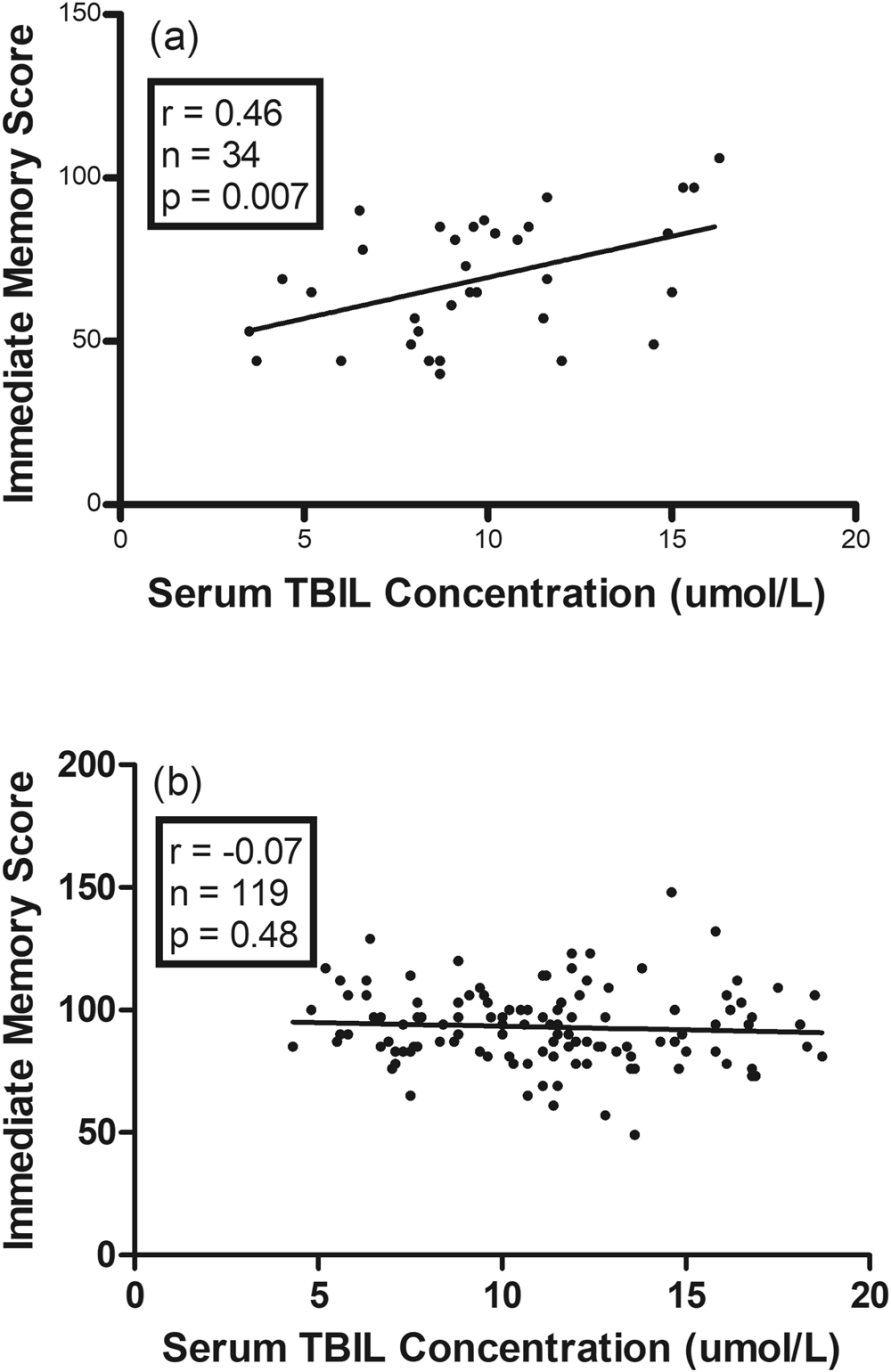
Correlation of serum TBIL concentration with the immediate memory score in SCH patients (**a**) and HCs (**b**). A significant correlation was found in SCH patients (r = 0.46, n = 34, p = 0.007), but not in HCs (r = -0.07, n = 119, p = 0.48).

The Pearson’s correlation analysis found a linear correlation of serum TBIL concentration with the immediate memory score in SCH patients, but not in HCs, as shown in Fig. 2. There were no significant correlations between serum TBIL concentration and other cognitive scores in SCH patients and HCs, respectively. Moreover, there were no relationships between serum TBIL concentration and cognitive scores in HCs with higher TBIL concentration (>11.14 umol/L or >14.00 umol/L).

Stepwise multiple regression analysis further indicated that serum TBIL concentration in SCH patients was related to the immediate memory score. In contrast, serum TBIL concentration in SCH patients was not associated with other cognitive scores (i.e., subscales of visuospatial/constructional, attention, language, and delayed memory, and RBANS total score). Moreover, serum TBIL concentration was not associated with the cognitive scores in HCs.

## Discussion

Our study showed that SCH patients had more severe cognitive impairment than HCs in most cognitive domains, except for the visuospatial/constructional domain, which is in line with our previous findings^9,10,42^. This finding is supported by several longitudinal and cross-sectional studies in first-episode SCH patients^43–45^. Interestingly, cognitive impairment has been found to be closely associated with the abnormality of leukoaraiosis that was influenced by the decreased BIL concentration in a sample of Korean adults^38–41^. Specifically, the change in BIL concentration may lead to microglia and astrocyte activation, myelination damage, and apoptosis of neuron cells in the brain^37^. These findings suggest that BIL might play a critical role in cognitive impairment. In the present study, serum TBIL concentration was positively correlated with the immediate memory score in SCH patients in a Chinese population. It is hypothesized that the immediate memory impairment may be mediated through antioxidant system defects resulting from decreased TBIL concentration^26,27,46,47^. A recent study has found a positive correlation of serum TBIL concentration with the cognitive score in MCI^35^. Serum concentration of BIL also was significantly associated with the recent memory score in SIVD patients^36^. Thus, the underlying mechanism responsible for the association of serum TBIL concentration with the immediate memory score could reflect the abnormality of antioxidant system that could further lead to neuronal and microglia impairment of SCH brain. Also, future studies should investigate the associations between other antioxidants and immediate memory score in first-episode SCH patients to confirm our findings.

Moreover, our findings indicated that serum TBIL concentration was significantly lower in SCH patients than that in HCs, which is consistent with previous studies^18–22^. Another study found that serum BIL concentration in HCs was approximately two-fold that in SCH patients^23^. Further studies have found that first-episode SCH patients had lower plasma TBIL concentration than HCs^22,23^. Compared to HCs, serum TBIL concentration also was significantly decreased in patients with major depressive disorder^48^. These findings further support the hypothesis that the defects of antioxidant defense system might be involved in the etiology of SCH^26,27^.

Several limitations should be noted in this study. *First of all, our study had a relatively small sample size*. These findings should be considered as preliminary. *Second, it was a cross-sectional study design*. Prospective studies with longitudinal follow-ups should be performed to confirm the association between serum TBIL concentration and immediate memory score in SCH patients. Thus, it is not clear whether there is a causal association of decreased serum TBIL concentration with the immediate memory deficits in SCH patients in this study. Furthermore, we did not differentiate direct and indirect BIL from TBIL because direct BIL was not measured. Additionally, although SCH patients were antipsychotic free for at least two weeks prior to this study, the effect of antipsychotics on serum BIL concentration could not be ruled out. Previous studies have indicated that antipsychotics and metabolic syndrome influence BIL concentration in SCH patients^49,50^. Thus, future studies should be performed in first-episode and drug-free SCH patients without metabolic syndrome. *Finally, we did not collect other relevant data, including body mass index, alcohol intake, and smoking*. Whether they might affect serum TBIL concentration and cognitive function in SCH patients, which should be further investigated in the future.

Collectively, we found that serum TBIL concentration was lower in SCH patients than that in HCs in a Chinese population. Serum TBIL concentration was positively associated with the immediate memory score in SCH patients. Our data further demonstrated that decreased TBIL concentration might reflect oxidant defense defects underlying the psychopathology of SCH. Also, the decline in serum TBIL concentration might play an important role in the immediate memory impairment in SCH patients. However, these findings should be regarded as preliminary due to little sample size, missing related data of antipsychotics use and metabolic syndrome, and absence of a longitude design. Thus, future studies should confirm our present findings in first-episode and drug-free SCH patients in a larger prospective sample.

## Methods

### Subjects

The sample size was calculated by an online website (http://powerandsamplesize.com/) according to the findings of previous studies^51,52^. The minimum sample size of this study was calculated for 32 SCH patients and 46 HCs. However, 34 SCH patients and 119 HCs (about 1:4 ratio) recruited in this study were further determined according to the previous study reporting 1: 4 case/control ratio for matched case-control studies to find valid associations with regards to the power^53^.

We recruited 34 SCH patients (male = 21, female = 13) at the inpatient unit of the Affiliated Guangji Hospital of Soochow University. The inclusion criteria included the following: (1) age between 18 and 60 years, Han Chinese; (2) diagnosis of schizophrenia as confirmed by two psychiatrists using the Structured Clinical Interview for DSM-IV (SCID); (3) no previous exposure to antipsychotics at least 2 weeks prior to the beginning of this study; 4) able to provide the signed informed consent and participate in the psychopathology assessment.

For comparison, 119 HCs (male = 71, female = 48) were recruited at the same time from Suzhou local community. They were all Han Chinese. Two psychiatrists assessed their present psychiatric status, and collected personal or family history of psychiatric disorders using unstructured interviews. They did not have any psychiatric disorders as well as family history of psychiatric disorders.

A complete medical history and physical examination were obtained from all subjects. Any subjects with other medical illness i.e. substance abuse/dependence, liver disorder, cancer, diabetes, and pregnancy were excluded. The Institutional Review Board of the Affiliated Guangji Hospital of Soochow University approved the informed consent and protocol of this study, and all experiments would be carried out in accordance with the approved guidelines and regulations. All subjects must sign informed consent before they participated in this study.

### Clinical measure

Each subject filled out a detailed questionnaire that recorded the general information, sociodemographic characteristics, and medical conditions. Other information was obtained from the available electronic medical records.

A clinical researcher used the RBANS to assess cognitive function in all subject^54^. The RBANS includes 12 subtests that are used to calculate a total score and 5 age-adjusted index scores. The test indices consist of attention, immediate memory, visuospatial/constructional, delayed memory, and language. Our research group has previously translated the English version of the RBANS into Chinese, and established its clinical validity and test-retest reliability in HCs and SCH patients^55^. The RBANS index scores were the standardized scores in this study.

Two psychiatrists used the PANSS to assess the severity of SCH psychopathology^56^. They had simultaneously attended a training session in the PANSS use before the beginning of this study. After training, a correlation coefficient greater more 0.8 was maintained for the PANSS total score by repeated assessments.

### TBIL measurement

Serum samples were collected from the forearm vein between 7 and 9 AM following an overnight fast. The serum of samples was separated, aliquoted, and stored at −80  °C in a refrigerator before serum TBIL concentration was measured. We used a HITACHI automatic biochemistry analyzer (Mode: 7180, Japan) to measure serum TBIL concentration through the method of chemical oxidation using a commercially available kit (MedicalSystem Biotechnology, China). The sensitivity of this kit was 3.42 umol/L, and its intra- and inter-assay variation coefficients were 4% and 5%, respectively. For each sample, serum TBIL concentration was assessed in triplicate by our technician.

### Statistical analysis

Since the sociodemographic and clinical characteristics including serum TBIL concentration were normally distributed in SCH patients and HCs (1-Sample Kolmogorov-Smirnov test), the comparisons between the two groups with regard to the sociodemographic and clinical characteristics were performed using analysis of variance (ANOVA) and Chi-squared test. The potential confounding variables were added as the covariates while the significant differences in the RBANS scores and serum TBIL concentration were observed between the two groups. Bonferroni correction were applied to each test to adjust for potential Type I error inflation due to multiple testing. Pearson’s correlation analyzed the correlations of serum TBIL concentration with the PANSS scores in SCH patients, and the associations of serum TBIL concentration with the RBNAS scores in SCH patients and HCs, respectively. Further stepwise multiple regression analysis was used to confirm the potential variables that might affect the RBANS scores in SCH patients and HCs. All statistical analyses were performed using the SPSS 17.0 for windows (SPSS Inc., Chicago, USA). The continuous variables were shown as mean and standard deviation (mean ± SD), and the statistical significance of all p values was set at 0.05 (2-sided).
